# Time-Space Clustering of Human Brucellosis, California, 1973–1992 ^1^

**DOI:** 10.3201/eid0807.010351

**Published:** 2002-07

**Authors:** Geoffrey T. Fosgate, Tim E. Carpenter, Bruno B. Chomel, James T. Case, Emilio E. DeBess, Kevin F. Reilly

**Affiliations:** * University of California, Davis, California, USA; † California Animal Health and Food Safety Laboratory, Davis, California, USA; ‡ Department of Human Services, Portland, Oregon, USA; § California Department of Health Services, Sacramento, California, USA

## Abstract

Infection with *Brucella* spp. continues to pose a human health risk in California despite great strides in eradicating the disease from domestic animals. Clustering of human cases in time and space has important public health implications for understanding risk factors and sources of infection. Temporal-spatial clustering of human brucellosis in California for the 20-year period 1973–1992 was evaluated by the Ederer-Myers-Mantel, Moran’s I, and population-adjusted Moran’s I procedures. Cases were clustered in concentrated agricultural regions in the first 5-year interval (1973–1977). Time-space clustering of human brucellosis cases in California late in the 20-year study period may reflect the distribution of Hispanic populations. Public health programs in California should focus on educating Hispanic populations about the risk of consuming dairy products, such as soft cheeses, made from unpasteurized milk.

Brucellosis is associated with chronic debilitating infections in humans and reproductive failure in domestic animals (1–3). Person-to-person transmission of brucellae is extremely rare (2,4), and human infection may be an accidental expression of a more widespread problem in animals (5). *Brucella* species considered important agents of human disease are *B. abortus* (primary reservoir in cattle), *B. melitensis* (sheep and goats), and *B. suis* (swine) (6). *B. melitensis* and *B. suis* are considered more pathogenic for humans than *B. abortus* (7). Dogs are reservoirs of *B. canis*; human infection has been documented to result in disease (3,8,9) but does not constitute an important public health concern in the United States (2,10).

Control and eradication of brucellosis in domestic animals have important public health implications. Test-and-slaughter programs, in conjunction with vaccination, are the major method of control (11). Whole herd depopulation (12) can also be used when other methods have reduced disease prevalence to low levels. Livestock populations can be screened for brucellosis by serologic testing of individual animals (12–16) or by testing pooled samples such as bulk milk (12,14). Several vaccines are available for reducing infection in animal populations (11), thereby reducing transmission potential to humans.

Persons infected with *Brucella* spp. usually have signs and symptoms consistent with an influenzalike or septicemic illness, often with insidious onset. The symptoms and clinical signs most commonly reported are fever, fatigue, malaise, chills, sweats, headaches, myalgia, arthralgia, and weight loss (8,10,17,18). Fewer than 10% of human cases of brucellosis may be recognized and reported (19), likely because of this misleading clinical picture (2,8). The acute form of human brucellosis is characterized by an undulating fever, in addition to the signs and symptoms mentioned. Lack of appropriate therapy during the acute phase may result in localization of bacteria in various tissues and lead to subacute or chronic disease that can have serious clinical manifestations (6,8,10).

Most cases involving field strains of *Brucella* can be traced to domestic food animals (5), and the prevalence of disease in livestock reservoirs reflects its occurrence in humans. Commonly, *B. abortus* and *B. suis* infections are associated with certain occupational groups, including farm workers, veterinarians, and meatpacking employees (6). Human *B. melitensis* infections occur more frequently in persons who do not have these occupational exposures (10). Persons usually become infected with brucellae through direct contact with infected animals or their products. Unpasteurized milk and processed dairy foods from infected animals are the major source of infection for the general population (7,10), and infected carcasses are the source of infection for workers in the meatpacking industry (20–22). Veterinarians can acquire brucellosis from assisting births in infected animals, as well as through inadvertent exposure to *B. abortus* strain 19 vaccine (5). Airborne transmission of bacteria to humans has also been documented in clinical laboratories and abattoirs (21, 23). Protective clothing and careful handling of infected animals can reduce occupation-related brucellosis (6,24), and avoiding unpasteurized dairy products should prevent infection in the general population (20).

The epidemiologic pattern of human infection with brucellae has been changing in the United States since 1947, when the number of reported cases was the highest ever recorded (6,321 total; 4.4 cases/100,000 population) (19,20). This change has been attributed to implementation of the state-federal Cooperative Brucellosis Eradication Program in 1934 and widespread milk pasteurization (19). *B. abortus* infection was common in the general population and before 1960 was the most frequent cause of human brucellosis (18). The relative importance of occupational exposures, especially in abattoir workers, steadily increased during the 1950s to 1970s (9,18,20,21). *B. suis* became the most frequent isolate in human cases from the mid-1960s to the early 1970s (9,18,19). The epidemiologic pattern of human brucellosis in the United States since the early 1970s may have shifted from an occupation-associated disease involving *B. suis* to one more common in the general population (23, 25). This change may be attributed to the swine brucellosis eradication program implemented in 1961 (22) and increased reporting of human *B. melitensis* infection (8,23,25), which was considered to have been eradicated from U.S. sheep and goats in 1972 (4). Hispanic populations of California are at increased risk for *B. melitensis* infection, with imported soft cheese the most commonly reported vehicle of exposure (23,25–27).

The objective of our study was to evaluate time-space clustering of reported human brucellosis cases in California for the 20-year period from 1973 to 1992. Determination of high-risk zones for human brucellosis may be useful in focusing education programs and public health funding.

## Materials and Methods

Human brucellosis is a reportable disease in California; data from 1973 to 1992 were obtained through the Office of Statistics and Surveillance and the Veterinary Public Health Section, California Department of Health Services. Data included county of residence, year of diagnosis, and patients’ reported race and age. Data without personal identifiers were obtained for cases confirmed according to Centers for Disease Control and Prevention definitions (28). Basic descriptive data for reported cases of brucellosis from 1992 to 2001 were also obtained from the California Department of Health Services. California census information was downloaded from the Demographic Research Unit, California Department of Finance website, at http://www.dof.ca.gov/html/Demograp/druhpar.htm.

The 20-year study period was divided into four 5-year periods for calculating county-level human brucellosis incidence. The numerator for the incidence calculation was the total number of cases in residents of each county in the 5-year period. The denominator was the county population for the median year of the 5-year interval. Crude county-level incidences were calculated, as well as incidences adjusted for race and combined age and race. The data were adjusted for race by categorizing the total population into Hispanic and non-Hispanic segments and for age by grouping the population into <10-, 10–19-, 20–29-, 30–39-, 40–49-, 50–59-, and >60-year categories. Brucellosis incidence proportions were adjusted directly by using the population distribution of Sacramento County in 1990 as the standard. In brief, direct adjustment of proportions was done by first calculating the proportion of brucellosis in each specific age-race category for all counties. This proportion was then multiplied by the number of persons in the corresponding age-race category of the standard population (Sacramento County, 1990) to yield the expected number of cases. This expected number was summed over all age-race categories for each county and then divided by the total standard population (total population of Sacramento County, 1990) to yield the adjusted proportion.

Because of its insidious onset, the actual date of onset of *Brucella* infection is often difficult to determine retrospectively. Therefore, the date of diagnosis for each case, rather than onset of symptoms, was used for calculating incidence. Cases were considered to have originated in the patient’s county of residence for all calculations, although the person may not have become infected in that county. Data were not available to evaluate the effect of these assumptions on incidence calculations. ArcView Geographic Information System, version 3, (Redlands, CA) was used to visually represent spatial distribution of brucellosis cases and incidence proportions in California counties.

The Ederer-Myers-Mantel (EMM) procedure (29) was used to examine time-space clustering of human brucellosis cases in each California county during the 20-year study period. This one-sided test for clustering was implemented in an Excel spreadsheet program (Microsoft Corp., Redmond, WA). Separate race-specific analyses were also performed for cases in Hispanic and non-Hispanic residents. The EMM test is sensitive to departures from a static population over time and is therefore not recommended for situations involving more than five periods, as the baseline population may change. To account for this limitation, the 20-year period was divided into four 5-year periods in which the base population should not change meaningfully. This division of the period results in analysis of 232 counties (58´4) over 5 years each, instead of 58 counties over 20 years. Cases in Los Angeles County during 1978–1982, for instance, will not be linked to cases in Los Angeles before or after this 5-year period. Different periods for the same county are treated as completely independent.

The Moran’s I test for spatial clustering (30) was performed to evaluate distribution of incidence proportions of human brucellosis in California counties during the study period. Data were analyzed by RAMAS Cast, version 2.0 (Applied Biomathematics, Sebauket, NY). This statistical procedure examines values in adjacent areas (counties), is two-sided, and calculates a standard normal z-score in which a positive test statistic indicates a tendency toward a clustered distribution and a negative statistic a tendency toward a uniform (dispersed) distribution.

The Moran’s I test was performed independently for the four 5-year cumulative incidences of brucellosis in each California county during the study period. This analysis was also performed on the average incidence for each county during the entire 20-year period. Crude proportions as well as race- and age/race-adjusted proportions were analyzed.

A modification of the Moran’s I technique, the population-adjusted Moran’s I (I_pop_), which adjusts for the underlying population density in each area (31), was used to evaluate spatial clustering of reported human brucellosis cases in California. The procedure was performed by using RAMAS Cast (Applied Biomathematics). Similar to the unadjusted Moran’s I, this statistical method is two-sided and calculates a standard normal z-score; however, the I_pop_ test is based on the numerator (number of cases) separate from the denominator (population at risk). Therefore, I_pop_ cannot be used for adjusted proportions because the data needed are numerators and denominators, rather than proportions.

The I_pop_ analysis was done by using numerator and denominator information from all four 5-year cumulative incidences of brucellosis in each California county during the study period. The total incidence for each county during the entire 20-year period was analyzed similarly, with mean county populations as the denominator. Spatial clustering was evaluated for the total population, as well as for Hispanic and non-Hispanic population segments.

Similar I_pop_ analyses were performed for reported cases in Hispanic and non-Hispanic segments of the population specific for *B. abortus* and *B. melitensis* infection. The causative *Brucella* species was determined either through bacteriologic isolation or determination of reported animal contact. Bacterial isolation was not done for all reported cases, and often the *Brucella* species was not determined because of concern about exposure risks for laboratory personnel. Patients reporting cattle as the primary animal contact were classified as having disease due to *B. abortus* when the infecting species was not determined. Similarly, patients reporting contact with goats (or goat cheeses) were classified as having infection with *B. melitensis*. This classification was necessary because the *Brucella* species involved was not identified for many cases.

## Results

**Figure 1:**
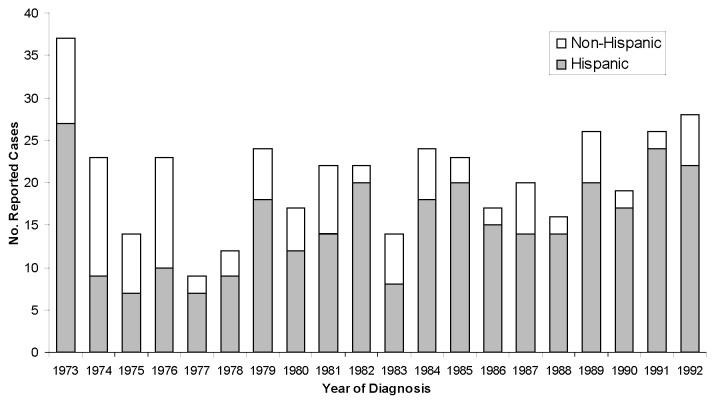
Number of reported cases of human brucellosis in Hispanic and non-Hispanic California residents, by year, 1973–1992.

**Figure 2:**
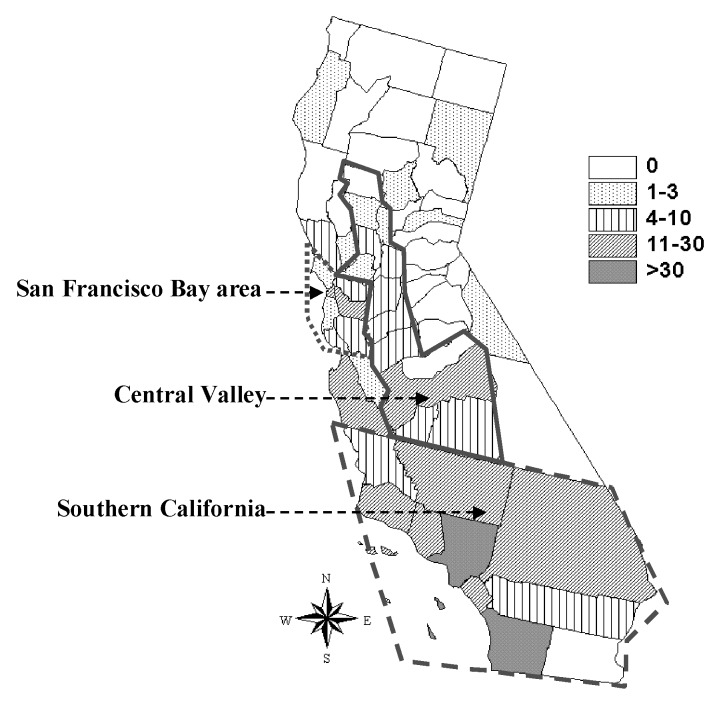
Distribution of reported cases of human brucellosis in California residents, 1973–1992.

A total of 426 human cases of brucellosis were reported in California from 1973 to 1992. Ten cases were excluded from analysis because recorded permanent residence was not in California, leaving 416 for study. Except for the period 1974–1976, the number of Hispanic cases reported each year from 1973 to 1992 was greater than the total for all other ethnic groups combined (Figure 1). Hispanics accounted for 305 (73%) of the reported cases in this 20-year period. The number of reported cases was highest in southern California (66%), with another 14% from the Central Valley and 12% from the San Francisco Bay area (Figure 2).

The *Brucella* species was identified in 229 (55%) of the 416 cases analyzed. *B. abortus* was isolated from 39 cases, *B. melitensis* from 181, *B. suis* from 9. Expanding classification to include information about contact with animal species led to determination of 91 cases due to *B. abortus* and 200 cases due to *B. melitensis*.

**Figure 3:**
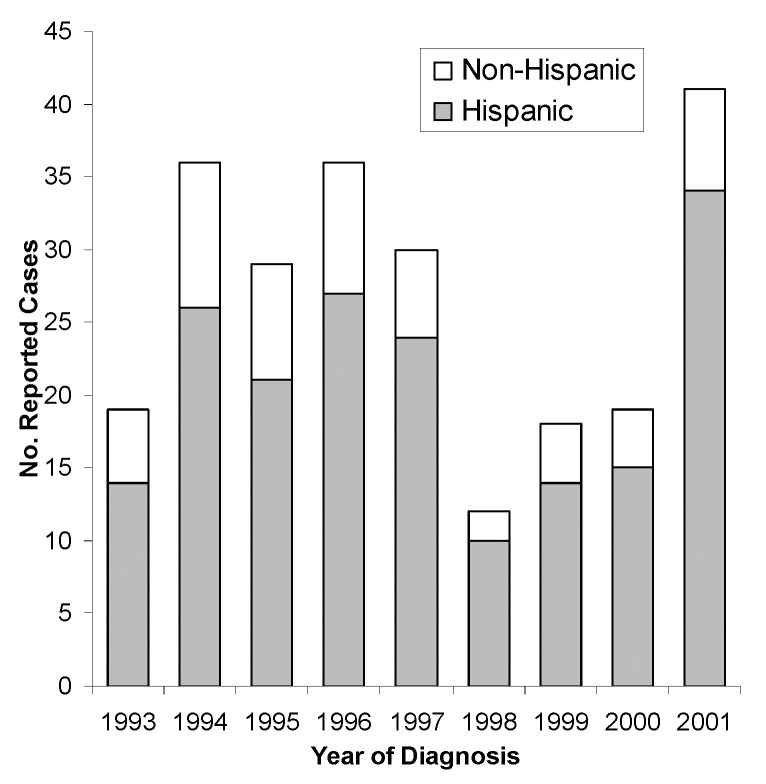
Reported cases of human brucellosis in Hispanic and non-Hispanic California residents, by year, 1993–2001.

Human brucellosis cases since 1992, reported mainly from southern California counties, also affected mostly Hispanics (Figure 3). Hispanics accounted for 185 (77%) of the 240 total cases reported in California from 1993 to 2001, which was similar to data for 1973–1992. Southern California counties accounted for 136 (57%) of the total cases; another 22% were from the Central Valley and 14% from the San Francisco Bay area. Counties reporting the largest number of cases were Los Angeles (53 cases), San Diego (30 cases), and Orange (19 cases).

According to the EMM procedure, clustering was statistically significant for Hispanic, non-Hispanic, and total cases (Table 1); this procedure was also used to determine relative contribution to overall clustering by individual county (Table 2). Most of the clustering effect during 1973–1982 in Hispanic cases was found in southern and Central Valley counties. Clustering was most pronounced in Los Angeles County, with 23 cases reported in 1973 although the maximum number of cases expected from the EMM procedure was 10 (chi square 74.9, p<0.001). During 1988–1992, substantial clustering of Hispanic cases also occurred in the San Francisco Bay area and southern California. Non-Hispanic cases were significantly clustered in the Central Valley and San Francisco Bay area during 1983–1987. Cases also clustered in southern California during 1988–1992.

**Table 1:** Ederer-Myers-Mantel (EMM) procedure for time-space clustering of reported human brucellosis cases, California, 1973–1992

Population	No. of cases	Chi square^a^	p value^b^
Hispanic	305	3.800	0.03
Non-Hispanic	111	6.078	0.007
Total	416	8.100	0.002

^a^EMM test statistic follows an approximate chi-square distribution with 1 degree of freedom. ^b^p-value based on a one-sided test (half of usual chi-square p-value).

**Table 2:** Ederer-Myers-Mantel (EMM) procedure for time-space clustering of reported human brucellosis cases, California, 1973–1992

Years	Population	County	California area^a^	Chi square	p value^b^
1973–1977	Hispanic	Los Angeles	Southern California	74.94	<0.001
		Orange	Southern California	4.57	0.02
		Stanislaus	Central Valley	2.99	0.04
1978–1982	Hispanic	San Luis Obispo	Southern California	5.83	0.008
		Santa Barbara	Southern California	3.31	0.03
		Fresno	Central Valley	2.99	0.04
		Kern	Southern California	2.99	0.04
1983–1987	Non-Hispanic	Sacramento	Central Valley	2.99	0.04
		Santa Cruz	San Francisco Bay	2.99	0.04
1988–1992	Hispanic	Alameda	San Francisco Bay	15.42	<0.001
		Ventura	Southern California	11.95	<0.001
	Non-Hispanic	Los Angeles	Southern California	4.57	0.02
		San Bernardino	Southern California	2.99	0.04

^a^Southern California = Imperial, Kern, Los Angeles, Orange, Riverside, San Bernardino, San Diego, San Luis Obispo, Santa Barbara, and Ventura Counties; Central Valley = Colusa, Fresno, Glenn, Kings, Madera, Merced, Sacramento, San Joaquin, Solano, Stanislaus, Sutter, Tulare, and Yolo Counties; San Francisco Bay = Alameda, Contra Costa, Marin, San Francisco, San Mateo, Santa Clara, and Santa Cruz Counties. ^b^p-value based on one-sided chi-square test with 1 degree of freedom.

**Figure 4:**
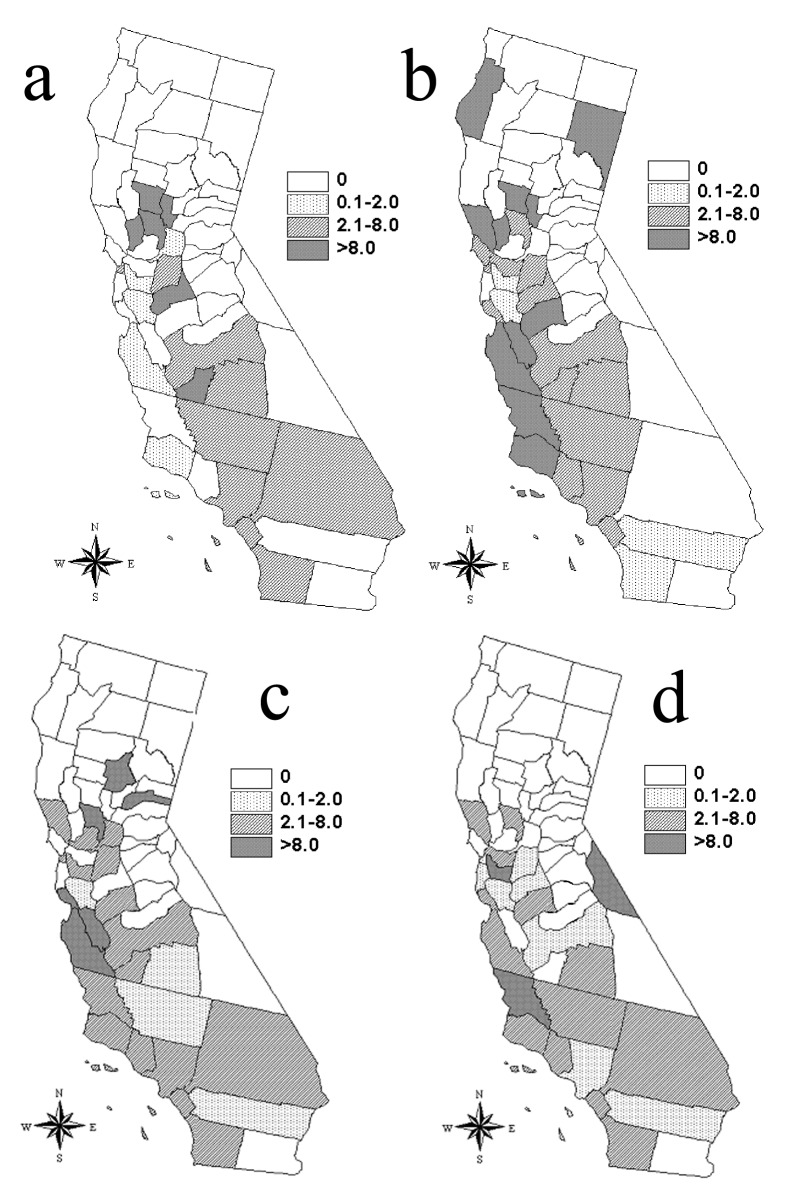
Distribution of human brucellosis: age/race-adjusted incidence per 10^6^ population in California for the following 5-year periods: (a) 1973–1977, (b) 1978–1982, (c) 1983–1987, and (d) 1988–1992.

The Moran’s I procedure demonstrated significant spatial clustering of crude incidence of human brucellosis in California for the 5-year periods from 1973 to 1977 and 1983 to 1987 (Table 3). Spatial clustering remained statistically significant for 1973–1977 after incidences were adjusted for differences in age and race structure of the California counties, but significance during 1983–1987 was removed by this adjustment (Table 3). The largest adjusted incidences of human brucellosis occurred in the Central Valley, the most active agricultural region of California, during 1973–1977 (Figure 4). Spatial distribution of large adjusted incidences shifted away from highly agricultural zones in the later periods, and clustering was not statistically significant.

**Table 3:** Moran’s I procedure for spatial clustering of the four, 5-year cumulative incidences of reported human brucellosis, California, 1973–1992

		Crude incidence^b^		Race adjusted^b^		Age-race adjusted^b^	
Years^a^	No. of cases	z-score^c^	p value	z-score^c^	p value	z-score^c^	p value
1973–1977	106	2.31	0.02	2.75	0.006	2.84	0.004
1978–1982	97	1.07	0.29	0.05	0.96	-0.34	0.74
1983–1987	98	4.62	<0.001	0.31	0.75	0.27	0.79
1988–1992	115	-0.32	0.75	-0.74	0.46	-0.49	0.63
1973–1992 (mean)	416	1.30	0.19	-0.56	0.57	-0.26	0.79

^a^Cases summed over the specified 5-year period. Incidence over the 20-year period is the mean of incidences for the 5-year periods. ^b^Incidences are based on the population of the 5-year period’s median year as the denominator. Incidences were adjusted by the direct method with the population distribution of Sacramento County in 1990 as the standard. Data for race were adjusted by using Hispanic and non-Hispanic population totals. ^c^Positive z-score indicates tendency towards clustering, negative value dispersion.

When the population as a whole was examined, the I_pop_ procedure showed substantial spatial clustering in all four 5-year study periods (Table 4). Significant clustering was also observed in the Hispanic-specific California population for all study periods except 1983–1987. Cases in the non-Hispanic population were not clustered during any period. The clustering effect in all tests resulted more from the number of cases within counties than from cases in adjacent counties (Table 4).

**Table 4:** Population-adjusted Moran’s I (I_pop_) analysis for spatial clustering of reported human brucellosis cases, California, 1973–1992

Years	No. of cases	z-score	p value	Within %^a^	Among %^a^
**Total population**					
1973–1977	106	7.79	<0.001	75.4	24.6
1978–1982	97	9.04	<0.001	62.1	37.9
1983–1987	98	4.94	<0.001	56.5	43.5
1988–1992	115	4.22	<0.001	93.1	6.9
1973–1992	416	18.67	<0.001	58.6	41.4
**Hispanic population**					
1973–1977	60	6.84	<0.001	77.6	22.4
1978–1982	73	11.66	<0.001	61.2	38.8
1983–1987	75	1.19	0.23	78.5	21.5
1988–1992	97	3.87	<0.001	107.9^b^	-7.9^b^
1973–1992	305	15.18	<0.001	70.8	29.2
**Non-Hispanic population**					
1973–1977	46	1.52	0.13	80.7	19.3
1978–1982	24	0.15	0.88	95.0	5.0
1983–1987	23	1.47	0.14	91.0	9.0
1988–1992	18	0.49	0.62	82.9	17.1
1973–1992	111	1.41	0.16	92.3	7.7

^a^Percentage of estimated spatial clustering attributed to cases in the same counties and in adjacent counties. ^b^All identified clustering attributed to cases in the same counties. Negative value in % demonstrates dispersion of cases in adjacent counties.

Significant spatial clustering was present in Hispanics infected with *B. abortus* during 1978–1982 (Table 5) and also in Hispanic populations infected with *B. melitensis* for all 5-year periods except 1983–1987. Cases of *B. abortus* or *B. melitensis* infection in non-Hispanic populations were not significantly clustered at any time during the 20-year study period.

**Table 5:** Population-adjusted Moran’s I (I_pop_) analysis for spatial clustering of reported human brucellosis cases due to *Brucella abortus* and *B. melitensis* in Hispanic populations, California, 1973–1992.

Years	No. of cases	z-score	p value	Within %^a^	Among %
***B. abortus*** ^b^					
1973–1977	12	-0.01	0.99	96.7	3.3
1978–1982	12	4.28	<0.001	74.8	25.2
1983–1987	9	-0.47	0.64	125.4^c^	-25.4^c^
1988–1992	23	0.15	0.88	108.8^c^	-8.8^c^
***B. melitensis***					
1973–1977	17	6.47	<0.001	62.4	37.6
1978–1982	48	8.77	<0.001	70.8	29.2
1983–1987	56	1.18	0.24	83.2	16.8
1988–1992	50	3.15	0.002	104.6^c^	-4.6^c^

^a^Percentage of estimated spatial clustering attributed to cases in same counties and in adjacent counties. ^b^Cases are reported as *B. abortus* or *B. melitensis* based on bacterial isolation or reported animal contact as cattle or goats, respectively, when bacterial isolation was not performed or species not determined. ^c^All identified clustering due to cases in the same counties. Negative value for in % demonstrates dispersion of cases in adjacent counties.

## Discussion

The Hispanic segment of the California population has been shown to be at a higher risk for brucellosis during the period from the early 1970s to the early 1990s (25). The number of cases reported during 1973–1992 was higher in Hispanics than in all other ethnic groups combined. Spatial distribution of cases was centered in southern California and other areas of the state with large Hispanic populations.

Results of the EMM procedure showed significant time-space clustering of reported human brucellosis cases during the study period. Human brucellosis is not considered a contagious disease (2,19); therefore, clustering could result from common-source outbreaks or time-space clustering of factors that increase risk of infection. Human brucellosis is often associated with work-related (18,21,22) and foodborne (5,27) outbreaks, both of which were reported in California during 1973–1992 (25). The EMM procedure demonstrated clustering in total cases as well as Hispanic and non-Hispanic segments of the population but could not distinguish between risk-factor and common-source causes. Clustering in the Hispanic population was most evident in Los Angeles and other southern California counties during the study period. Non-Hispanic cases also tended to cluster in southern California, but not to the same degree as observed for Hispanics.

Spatial clustering of brucellosis incidence found by the Moran’s I technique during 1973–1977 was significant even after the data were adjusted for age and race structure of county populations. This finding suggests that, during this 5-year period, clustering of cases did not result simply from spatial distribution of Hispanic populations. Until recently, brucellosis had been reported to be most strongly associated with occupation; farm workers, veterinarians, and meatpacking employees were at highest risk. Counties with the highest adjusted incidences during this period were those with a high degree of agricultural activity, suggesting the importance of traditional exposures.

Crude incidences of county-level human brucellosis were significantly clustered for 1983–1987 based on Moran’s I. However, adjusting proportions for underlying race distributions removed the clustering, suggesting that clustering of human brucellosis cases during this time reflected the distribution of the Hispanic population. These findings confirm other reports that the epidemiology of human brucellosis is shifting from a disease of certain occupational groups to a foodborne disease of the general population, with Hispanics at greatest risk (23,25).

Reported cases of human brucellosis were significantly clustered in all four 5-year periods based on total population I_pop_ analysis. Reported cases in Hispanics were also significantly clustered in all 5-year periods except 1983–1988. Results also show that most clustering was due to the number of cases within counties, rather than in adjacent counties. This finding was most apparent in the Hispanic-specific analysis for 1988–1992. More than 100% of the clustering effect was estimated for cases in the same counties. Cases in adjacent counties during this period were dispersed, driving the test statistic in the other direction (negative clustering effect). Clustering in specific counties was strong enough to overcome this dispersion effect. Brucellosis incidences in non-Hispanics of California were not clustered at any time during the study period.

The I_pop_ analysis suggests that occurrence of human brucellosis in non-Hispanics of California is a random event that does not appear to cluster in certain counties or regions. In contrast, Hispanic cases showed a strong tendency to be clustered in certain counties. This tendency was especially true for 1988–1992, when nearly 50% (48/97) of Hispanic cases were reported in three nonadjacent California counties: Los Angeles (23 cases), San Diego (14 cases), and Alameda (11 cases).

Identification of spatial clustering of human disease for specific *Brucella* species can provide important epidemiologic information about animal reservoir and source of infection. Human infection due to *B. abortus* would be expected to cluster in counties with increased livestock activity early in the study period, when *B. abortus* was still endemic on some farms. Lack of significant clustering for 1973–1977 may have resulted from the small number of confirmed *B. abortus* infections and the resulting low statistical power for spatial tests. Spatial clustering of *B. melitensis* associated with Hispanic populations would be expected to be consistent throughout the 20-year study period. This observation held true except for 1983–1987, when no significant clustering was found for brucellosis cases in Hispanics or for *B. melitensis*-specific cases.

Data were analyzed by both the unadjusted and population-adjusted Moran’s I techniques, because of known characteristics of human brucellosis. The I_pop_ analysis is more powerful than the unadjusted Moran’s I (31), so the I_pop_ procedure is recommended when both numerator and denominator data are available. However, the null hypothesis of this statistical procedure is that cases are independent occurrences of disease in the underlying population at risk. Rejection of this null hypothesis leads to acceptance of the alternate hypothesis that clustering in disease occurrence is present. Human brucellosis is known to cluster in occupational and foodborne settings. Therefore, the statistical test may be biased because it is not able to subtract the effect of these outbreaks from the overall test statistic. This bias could be controlled if data were available for all outbreaks that occurred during the study period. The unadjusted Moran’s I technique has lower power and is therefore more conservative than the I_pop_. The true nature of human brucellosis clustering in California during 1973–1992 most likely falls somewhere between the results of these two statistical analyses.

Human brucellosis continues to be a major public health concern in California even though the United States has effectively reduced the level of *Brucella* infection in domestic animals (32). Our results suggest that the epidemiology of risk factors for human infection due to *Brucella* spp. in California has changed. More traditional sources of infection are less important than the increased risk of *Brucella* spp. as foodborne pathogens. Traditional clustering of cases in concentrated agricultural regions was observed only for 1973–1977.

The Hispanic segment of the California population is at higher risk for disease due to *Brucella* infection than all other ethnic groups. Counties with high crude incidences of brucellosis correspond to those with large Hispanic populations. Increased risk has been attributed to certain dietary preferences, particularly for Mexican soft cheeses (23,25–27). Results suggest that time-space clustering of human brucellosis cases in California during the study period was predominantly due to clustered distribution of Hispanics in the state. However, I_pop_ and EMM results indicate residual temporal-spatial clustering of Hispanic cases in certain counties after the data were controlled for race through Hispanic-specific calculations. Counties with the highest adjusted incidences of human brucellosis during 1988–1992 were San Luis Obispo, Mono, and Alameda Counties. Only a single case was reported in Mono County during 1988–1992, but the small population size resulted in a relatively large incidence proportion.

Public health programs should focus on educating the Hispanic segment of the California population about the risks of consuming certain dairy products, such as soft cheeses, made from unpasteurized milk. Education must also extend to health-care providers who work in areas with large Hispanic populations likely to be exposed to illegally imported dairy products from areas where *Brucella* infection is still common in domestic animals. Efforts should be focused in southern California and San Francisco Bay area counties with the highest brucellosis incidences and absolute number of reported cases. More research focusing on the epidemiology of human brucellosis in California is necessary to aid in protection of its residents from disease.
