# Correction Vol. 8, No. 7

**DOI:** 10.3201/eid0808.C10808

**Published:** 2002-08

**Keywords:** Errata, erratum, correction

In Time-Space Clustering of Human Brucellosis, California, 1973-1992, by G. Fosgate et al., an error appears in the results section. The corrected sentence appears below and online at http://wwwnc.cdc.gov/eid/article/8/7/01-0351_article.htm.

The *Brucella* species was identified in 229 (55%) of the 416 cases analyzed. *B. abortus* was isolated from 39 cases, *B. melitensis* from 181, and *B. suis* from 9.

We regret any confusion this error may have caused.

